# Simultaneous liver-kidney transplantation: future perspective

**DOI:** 10.1007/s00345-024-05174-z

**Published:** 2024-08-20

**Authors:** Thomas Prudhomme, Benoit Mesnard, Julien Branchereau, Mathieu Roumiguié, Charlotte Maulat, Fabrice Muscari, Nassim Kamar, Michel Soulié, Xavier Gamé, Federico Sallusto, Marc Olivier Timsit, Sarah Drouin

**Affiliations:** 1 Department of Urology, Kidney Transplantation and Andrology, TSA 50032 Rangueil Hospital, Toulouse Cedex 9, 31059 France; 2 Center for Research in Transplantation and Translational Immunology, Nantes Université, INSERM, UMR 1064, Nantes, 44000 France; 3 Department of Urology, University Hospital of Nantes, Nantes, France; 4 Department of Digestive Surgery, University Hospital of Rangueil, Toulouse, France; 5 Department of Nephrology and Organ Transplantation, University Hospital of Rangueil, Toulouse, France; 6 Department of Urology, Hôpital Européen Georges Pompidou, APHP-Centre, Paris, France; 7 Service Médico-Chirurgical de Transplantation Rénale, APHP Sorbonne-Université, Hôpital Pitié-Salpêtrière, Paris, Île-de-France, France

**Keywords:** Simultaneous liver-kidney transplantation, Liver transplantation, End-stage renal disease, Hypothermic machine perfusion

## Abstract

**Background:**

The aims of this narrative review were (i) to describe the current indications of SLKT, (ii) to report evolution of SLKT activity, (iii) to report the outcomes of SLKT, (iv) to explain the immune-protective effect of liver transplant on kidney transplant, (v) to explain the interest of delay kidney transplantation, using hypothermic machine perfusion (HMP), (vi) to report kidney after liver transplantation (KALT) indications and (vii) to describe the value of the increase in the use of extended criteria donors (ECD) and particular controlled donation after circulatory death (cDCD) transplant, thanks to the development of new organ preservation strategies.

**Method:**

Electronic databases were screened using the keywords "Simultaneous", "Combined", "kidney transplantation" and "liver transplantation". The methodological and clinical heterogeneity of the included studies meant that meta-analysis was inappropriate.

**Results:**

A total of 1,917 publications were identified in the literature search. Two reviewers screened all study abstracts independently and 1,107 of these were excluded. Thus, a total of 79 full text articles were assessed for eligibility. Of these, 21 were excluded. In total, 58 studies were included in this systematic review.

**Conclusions:**

Simultaneous liver-kidney transplantation has made a significant contribution for patients with dual‐organ disease. The optimization of indication and selection of SLKT patients will reduce futile transplantation. Moreover, increasing the use of transplants from extended criteria donors, in particular cDCD, should be encouraged, thanks to the development of new modalities of organ preservation.

**Supplementary Information:**

The online version contains supplementary material available at 10.1007/s00345-024-05174-z.

## Introduction

Liver transplantation (LT) is an established treatment for patients with end-stage liver disease (ESLD) and acute liver failure. End-stage renal disease (ESRD) is common and increases mortality in wait-listed LT patients [1, 2]. ESRD in LT recipients is responsible of worse patient survival and outcomes [3–6]. Thus, ESRD has long been considered a contraindication to liver transplantation [2].

Simultaneous liver-kidney transplantations (SLKT), first reported in 1984 by Margreiter et al. [7] has been developed for patients with ESLD and ESRD, with optimal short-term and long-term outcomes [8]. In 2002, in the US, the *model for end-stage liver disease* (MELD) scoring system was introduced for organ allocation for wait-listed LT patients [9]. This scoring system includes the renal function of the LT candidates. Thus, since that time, the incidence of ESRD among LT recipients increased, resulting in an increase in SLKT activity since 2002 [5, 10, 11].

The aims of this narrative review were (i) to describe the current indications of SLKT, (ii) to report evolution of SLKT activity, (iii) to report the outcomes of SLKT, (iv) to explain the immune-protective effect of liver transplant on kidney transplant, (v) to explain the interest of delay kidney transplantation, using hypothermic machine perfusion (HMP), (vi) to report kidney after liver transplantation (KALT) indications and (vii) to describe the value of the increase in the use of extended criteria donors (ECD) and particular controlled donation after circulatory death (cDCD) transplant, thanks to the development of new organ preservation strategies.

## Methods

### Methodology

This article is a narrative review on simultaneous liver-kidney transplantation with the aim to describe the current indications of SLKT, to report evolution of SLKT activity, to report the outcomes of SLKT, to explain the immune-protective effect of liver transplant on kidney transplant, to explain the interest of delay kidney transplantation, using hypothermic machine perfusion (HMP), to report kidney after liver transplantation (KALT) indications and then to describe the value of the increase in the use of extended criteria donors (ECD) and particular controlled donation after circulatory death (cDCD) transplant, thanks to the development of new organ preservation strategies.

### Search strategy and study selection

This narrative review has been conducted according to Preferred Reporting Items for Systematic Reviews and Meta-analyses (PRISMA) statement [12]. Studies (January 1, 1995 to April 31, 2023) were identified by highly sensitive searches of electronic databases (Embase, Medline, Cochrane Library databases, PubMed). The search was complimented by additional sources including the reference lists of included studies. Full strategy research is show in the Supplementary Table 1. All study designs were eligible for inclusion, except letter to the editor, case reports and studies published as conference abstracts only. Only studies published in 1995 and after were included to reflect current clinical practice. Language was restricted to English and French pragmatic reasons. Titles and abstracts of all identified studies were independently reviewed by two authors (T.P., B.M.) and discrepancies were resolved by a third reviewer (S.D.). The methodological and clinical heterogeneity of the included studies meant that meta-analysis was inappropriate. Therefore, a narrative synthesis of the data was performed.

A total of 1,917 publications were identified in the literature search. Two reviewers (T.P., B.M.) screened all study abstracts independently and 1,107 of these were excluded. Thus, a total of 79 full text articles were assessed for eligibility. Of these, 21 were excluded. In total, 58 studies were included in this systematic review (Supplementary Fig. 1).

### Current indications of SLKT

The indications for SLKT can be divided into three categories [2, 13]:

(i) ESLD with chronic kidney disease, including: glomerulonephritis, interstitial renal disease, polycystic disease, and calcineurin inhibitors (CNI) toxicity;
(ii) ESLD with acute kidney injury, including: hepatorenal syndrome (HRS) and acute tubular necrosis;
(iii) Metabolic disorders, including: primary hyperoxaluria I, alpha 1 antitrypsin deficiency, glycogen storage disease type I, sickle cell disease, amyloidosis, haemolytic uraemic syndrome, methylmalonic acidemia.

**Fig. 1:**
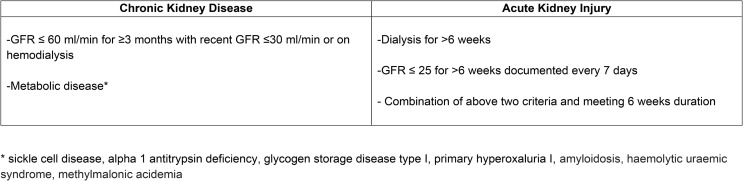
OPTN 2017 criterion for simultaneous liver-kidney transplantation

In 2017, an allocation policy for SLKT was created in the US, which defines eligibility criteria for SLKT [11, 14] (Fig. 1). Currently, SLKT indication is consensual for ESLD with chronic kidney disease or metabolic disorder but it remains a cause for debate for ESLD with acute renal failure, including hepatorenal syndrome (HRS). HRS is a functional acute kidney injury secondary to acute or chronic liver failure [13, 15, 16]. HRS is reversible as shown by the successful transplantation of affected kidneys from patients dying of liver failure with HRS to patients with normal liver function [17] and renal recovery following LT in patients with HRS [18]. However, patients with prolonged HRS may not recover renal function with LT alone and may require SLKT or kidney after LT (KALT) [19].

### Evolution of SLKT activity

In 2002, the *model for end-stage liver disease* (MELD) scoring system was created in the US, (which prioritizes renal dysfunction) resulting in an over 400% increase of SLKT between 2001 (138 SLKT; 2.5% of LT) and 2016 (738 SLKT; 9.3% of LT) [9, 20]. Prior to introduction of the MELD system, SLKT accounted for only 1.7% of LT activity in 1990 in the US. This growing number of SLKT is not only a consequence of the MELD allocation system, but likely also because of changing practices in transplant centers [21]. In 2017, an UNOS allocation policy for SLKT was created in the US, which defines eligibility criteria for SLKT [11, 14]. Indeed, before this period, each transplant center decide the SLKT eligibility on a case-by‐case basis [2]. From the application of UNOS allocation policy, a progressive decrease of SLKT activity between 2016 and 2019 (738 SLKT in 2016 versus 704 SLKT in 2019) has been reported in the US [22].

In France, SLKT activity increased between 2010 (40 SLKT) and 2015 (74 SLKT) but in a lower proportion than in the US. Thus, in 2021, 58 (SLKT) were performed in France, accounted for 4.8% (58/1204) [23] of LT activity [23].

Indeed, in France, the allocation of LT is performed according to 3 main modalities: super-emergency, Liver score (since 2007) and “out of round”. Thus, Liver score considers the indication, the severity of the patient’s condition (MELD score in the case of cirrhosis, Alpha-fetoprotein score for hepatocarcinoma), the waiting time and the distance between the procurement and transplantation sites [24]. Therefore, ESRD has less impact on the LT indication.

In UK, this increase in SLKT activity has also been limited. Indeed, from January 2001 to December 2013, 5912 (98%) patients received a liver transplant alone and 123 (2%) a SLKT [25].

### SLKT outcomes: patient and graft survival

National US transplant registry data and single center data analyses reported a 5-year patient survival rates after SLKT range from 64 to 76% [9, 26–29].

Patients with criteria for SLKT (Fig. 1) have better outcomes after receiving SLKT compared to LT alone recipients [28, 29]. Indeed, Hmoud et al. [28] reported patient survival, from the UNOS national database, comparing LT alone (with ESRD and wait-listed for KT) versus SLKT. They reported a significant higher 5-year patient survival in SLKT group (76% versus 55%). Similarly, Fong et al. [29] reported a significant higher 5-year patient survival in SLKT group, compared to LT alone (67.4% versus 62.9%).

In case of ESLD with acute renal failure, the etiology of acute renal failure impacts the LT outcomes. Indeed, Nadim et al. [30] compared LT outcomes, performed in acute renal failure patient, according to acute renal failure causes (HRS or acute tubular necrosis). They reported significant lower 5-year patient survival after LT in case of acute tubular necrosis (< 45% versus 75–80%).

Tinti et al. [25] reported the national UK experience on SLKT. Thus, from January 2001 to December 2013, 5912 (98%) patients received a LT and 123 (2%) a SLKT. They compared patient and graft survival between SLKT and LT, after stratifying on pretransplant estimated glomerular filtration rate (eGFR). They reported similar 5-year patient and graft survival between SLKT and LT, whatever pretransplant eGFR. However, they reported a significant higher patient survival, for patients on dialysis at time of transplantation, in SLKT group.

### Immune-protection by the liver transplant in SLKT. Immunological aspects

In their study of the UNOS database, Fong et al. [31] and Simpson et al. [32] reported a lower incidence of kidney acute rejection and a higher kidney allograft survival in SLKT, as compared to KT alone. Indeed, the liver graft among SLKT recipients ensure an immune-protection to the kidney grafts, particularly in case of preformed donor-specific anti-HLA antibodies (DSA) [33–36].

Several mechanisms have been proposed, to explain the immunologic protection afforded by the liver to the kidney, which include adsorption of preformed DSA and removal by Kupffer cells and production by the liver allograft of soluble HLA class I antigens which can neutralize both pre-existing DSA and cytotoxic T lymphocytes [37].

### Simultaneous or delayed KT, using hypothermic machine perfusion (HMP), after LT?

Studies from UNOS database reported a significant lower kidney transplant survival and higher kidney transplant primary non-function for SLKT in high risk medical patients (MELD scores > 30), compared to recipients with MELD scores < 30 [38].

The lower kidney transplant survival observed for SLKT recipients with MELD score > 30 is caused by the physiological disturbances generated by LT [39, 40]. Indeed, recipients of LT often require vasopressors to correct perioperative hypotension. This perioperative hypotension and vasopressors are harmful for kidney allograft [41], resulting in a lower kidney transplant survival.

In order to limit these detrimental effects on kidney allograft function, several teams reported their experience of delay of kidney transplantation [39, 40, 42, 43]. Thus, kidney transplants were preserved with hypothermic machine perfusion (HMP), to defer the KT until stabilization of the coagulopathy and hemodynamic status of the recipients.

Ekser et al. [39] compared the outcomes of simultaneous and delayed KT in SLKT. They included 69 simultaneous KT and 61 delayed KT. As expected, mean kidney cold ischemia time was longer in delayed KT group (10 and 50 h). They reported a significant higher delayed graft function (DGF), lower 1-year eGFR and higher 1-year and 5-year patient survival in delayed KT group. These results are confirmed by Lunsford et al. [40] in their multicentric retrospective study. From February 2004 to January 2017, they included 63 delayed KT and 161 simultaneous KT. They reported a significant higher 1-year, 3-year and 5-year patient and kidney transplant survival in delayed KT group.

Recently, Chang et al. [43] reported kidney outcomes of HMP versus static cold storage of kidney grafts in SLKT, from UNOS database. Kidney cold ischemia time was longer in the HMP group (12.8 h versus 10.0 h). They reported that HMP reduced DGF but not PNF.

### Kidney after liver transplantation (KALT) or SLKT?

KALT was the historical indication of LT in recipients who develop ESRD due calcineurin inhibitor-induced nephrotoxicity. KALT, in an early stage after LT, has the advantage of a lower perioperative morbidity and greater supply of kidneys (deceased or living donor kidney grafts). Simpson et al. [32] compared outcomes of KALT and SLKT, from the UNOS national database. From 1996 to 2003, they included 352 KALT and 1,136 SLKT. They reported a significant shorter kidney transplant half-life and a lower rejection-free kidney transplant survival after KALT, compared to SLKT. Recently, Cullaro et al. [44] reported data on kidney fonction and survival comparing SLKT (according to MELD score) and KALT, also from the UNOS national database. They reported a higher risk of early kidney failure in SLKT recipients with an MELD score ≥ 25, as compared to KALT recipients. Thus, KALT should probably be considered in patients with high MELD score.

### Extended criteria donor and donation after circulatory death (DCD) transplant. A way to increase the SLKT pool?

Main studies comparing donation after brain death (DBD) and cDCD SLKT are summarized in Tables 1, 2 and 3.

**Table 1:** Studies evaluating cDCD SLKT and LT outcomes: studies, recipients and donors characteristics

Author, date	Study type	Inclusion period	Arms (*n*° of recipients and donor type)	Recipients age (years)	Recipient sex (% of male)	Preemptive KT (%)	Median biologic MELD	Retransplant rate (%)	Donor age (years)	Donor sex (% of male)
**STUDIES EVALUATING cDCD VERSUS DBD IN SLKT**										
Nunez-Nateras et al., 2020 [45]	Retrospective cohort study	2010–2018	30 cDCD SLKT	57.1	53.3	36.7	24.2	Liver: 6.7 Kidney: 10	37.4	60.0
			131 DBD SLKT	54.8	59.5	26.0	28.9	Liver: 16 Kidney: 9.2	37.8	56.5
Croome et al., 2020 [46]	Retrospective UNOS cohort study	2000–2010 (Era 1)	94 cDCD SLKT	53.7	66.0	*	27.6	Liver: 9.6	32.3	*
		2011–2018 (Era 2)	208 cDCD SLKT	56.7	63.0	*	27.8	Liver: 2.4	33.0	*
			624 DBD SLKT (propensity matched)	56.1	63.8	*	27.7	Liver: 2.2	34.8	*
LaMattina et al., 2011 [47]	Retrospective cohort study	1998–2008	5 cDCD SLKT	47.0	*	*	33.0	*	35.0	*
			32 DBD SLKT	54.4	*	*	25.0	*	45.5	*
**STUDIES EVALUATING NRP VERSUS SRR IN cDCD LT**										
Hessheimer et al., 2019 [60]	Retrospective cohort study	2012–2016	95 NRP cDCD LT	54.8	77.9	/	15.1	2.1	53.8	66.3
			117 SRR cDCD LT	57.7	84.6	/	14.1	1.7	54.5	65.8
Watson et al., 2019 [51]	Retrospective UK national database cohort study	2011–2017	43 NRP cDCD LT	60.0	*	/	15	*	41.0	*
			187 SRR cDCD LT	57.0	*	/	15	*	50.0	*
Hessheimer et al., 2022 [50]	Retrospective cohort study	2012–2019	545 NRP cDCD LT	59.0	79.0	/	12.0	3.1	59.0	64.0
			258 SRR cDCD LT	58.0	83.0	/	12.0	1.2	58.0	65.0
**STUDIES EVALUATING NRP cDCD VERSUS DBD IN LT**										
Savier et al., 2020 [52]	Retrospective French national database cohort study	2015–2019	50 NRP cDCD LT	59.9	*	/	7	*	50.0	68.0
			100 DBD LT	58.4	*	/	10	*	50.0	61.0
**STUDIES EVALUATING NP VERSUS SCS IN LT**										
Nasralla et al., 2018 [56]	Prospective randomized controlled trial	2014–2016	170 NP LT (107 DBD and 63 cDCD)	55.0	71.1	/	13.0	Liver: 9.9	56.0	59.1
			164 SCS LT (104 DBD and 60 cDCD)	55.0	73.3	/	14.0	Liver: 7.9	56.0	57.1
**STUDIES EVALUATING HOPE VERSUS SCS IN LT**										
Schlegel et al., 2019 [61]	Retrospective cohort study	2012–2017	50 HOPE cDCD LT	58.0	*	/	11.0	*	57.0	*
			50 SCS cDCD LT	57.0	*	/	11.8	*	53.0	*
			50 SCS DBD LT	57.0	*	/	15.0	*	50.0	*
Czigany et al., 2021 [62]	Prospective randomized controlled trial	2017–2020	23 HOPE ECD DBD LT	60.0	78.0	/	13.0	*	73.0	52
			23 SCS ECD DBD LT	63.0	87.0	/	17.0	*	71.0	56
Schlegel et al., 2023 [57]	Prospective randomized controlled trial	2015–2019	85 HOPE DBD LT	60.0	64.7	/	20.0	5.9	59.0	52.9
			85 SCS DBD LT	57.0	78.8	/	19.0	2.4	62.0	50.0
**STUDIES EVALUATING NRP VERSUS HOPE IN cDCD LT**										
Muller et al., 2020 [58]	Retrospective cohort study	2012–2019	132 NRP cDCD LT	59.5	*	/	12.0	*	50.0	*
			93 HOPE cDCD LT	59.0	*	/	12.0	*	61.0	*
**STUDIES COMPARING NRP VERSUS NP IN cDCD LT**										
Mohkam et al., 2022 [63]	Retrospective cohort study	2015–2019	34 NP cDCD LT	56.0	52.9	/	12.0	*	48.0	58.8
			68 NRP cDCD LT	57.0	85.3	/	12.0	*	49.0	70.6
Gaurav et al., 2022 [59]	Retrospective cohort study	2013–2020	69 NRP cDCD LT	56.0	70.0	/	14.0	12.0	51.0	65.0
			67 NP cDCD LT	59.0	67.0	/	14.0	5.0	52.0	58.0
			97 SCS cDCD LT	56.0	58.0	/	16.0	1.0	50.0	53.0

SLKT Simultaneous Liver-Kidney Transplantations; LT: Liver Transplantation; KT: Kidney Transplantation; DBD: Donation after Brain Death; cDCD: Controlled Donation after Circulatory Death; NP: Ex-Situ Normothermic Perfusion; NRP: Normothermic Regional Perfusion; SCS: Static Cold Storage; SRR: Super-Rapid Recovery; HOPE: Ex-Situ Hypothermic Oxygenated Perfusion; ECD: Extended Criteria Donors

**Table 2:** Studies evaluating cDCD SLKT and LT outcomes: intra-operative and post-operative outcomes

Author, date	Arms (Number of recipients and donor type)	NRP use (% and minutes)	WIT (minutes)	Liver CIT (hours)	Kidney CIT (hours)	Delayed graft function (%)	Primary non function (%)	1-year creatinine (mg/dL)	1-year eGFR (ml/min)	Dialysis at 1-year (%)
**STUDIES EVALUATING cDCD VERSUS DBD IN SLKT**										
Nunez-Nateras et al., 2020 [45]	30 cDCD SLKT	0%	22.0	5.3	8.2	40	Liver: 3.3 Kidney: 0	1.36	57.7	3.3
	131 DBD SLKT	/	/	5.8	8.2	23.7	Liver: 0.8 Kidney: 3.8	1.37	56.3	1.5
Croome et al., 2020 [46]	94 cDCD SLKT (Era 1)	0%	17.1	6.8	11.5	*	*	*	*	*
	208 cDCD SLKT (Era 2)	0%	17.0	6.4	10.3	*	*	*	*	*
	624 DBD SLKT (propensity matched) (Era 2)	/	/	6.4	11.4	*	*	*	*	*
LaMattina et al., 2011 [47]	5 cDCD SLKT	0%	19.0	4.6	12.5	80.0	Liver: 0 Kidney: 0	*	*	*
	32 DBD SLKT	/	/	8.0	12.5	31.0	Liver: 6.3 Kidney: 3.1	*	*	*
**STUDIES EVALUATING NRP VERSUS SRR IN cDCD LT**										
Hessheimer et al., 2019 [60]	95 NRP cDCD LT	100% 120 min	19.2	5.6	/	/	2.0	*	*	*
	117 SRR cDCD LT	0%	23.1	5.7	/	/	3.0	*	*	*
Watson et al., 2019 [51]	43 NRP cDCD LT	100% 123 min	30.0	6.4	/	/	0	*	72	*
	187 SRR cDCD LT	0%	27.0	7.4	/	/	7.0	*	70	*
Hessheimer et al., 2022 [50]	545 NRP cDCD LT	100% 111 min	12.0	5.3	/	/	3.0	*	*	*
	258 SRR cDCD LT	0%	14.0	5.6	/	/	6.0		**	*
**STUDIES EVALUATING NRP cDCD VERSUS DBD IN LT**										
Savier et al., 2020 [52]	50 NRP cDCD LT	100% 190 min	*	5.8	/	/	*	*	*	*
	100 DBD LT	/	/	6.3	/	/	*	*	*	*
**STUDIES EVALUATING NP VERSUS SCS IN LT**										
Nasralla et al., 2018 [56]	170 NP LT (107 DBD and 63 cDCD)	0%	21.0	2.1	/	/	0.8	*	*	*
	164 SCS LT (104 DBD and 60 cDCD)	0%	16.0	7.8	/	/	0	*	*	*
**STUDIES EVALUATING HOPE VERSUS SCS IN LT**										
Schlegel et al., 2019 [61]	50 HOPE cDCD LT	0%	31.0	4.4	/	/	0	*	*	*
	50 SCS cDCD LT	0%	17.0	4.7	/	/	4.0	*	*	*
	50 SCS DBD LT	/	/	5.0	/	/	2.0	*	*	*
Czigany et al., 2021 [62]	23 HOPE ECD DBD LT	/	/	8.3	/	/	4.0	*	*	*
	23 SCS ECD DBD LT	/	/	8.4	/	/	4.0	*	*	*
Schlegel et al., 2023 [57]	85 HOPE DBD LT	/	/	6.2	/	/	0	*	*	*
	85 SCS DBD LT	/	/	7.1	/	/	3.5	*	*	*
**STUDIES EVALUATING NRP VERSUS HOPE IN cDCD LT**										
Muller et al., 2020 [58]	132 NRP cDCD LT	100% 184 min	22.0	5.7	/	/	2.3	*	*	*
	93 HOPE cDCD LT	0%	31.0	6.4	/	/	4.3	*	*	*
**STUDIES COMPARING NRP VERSUS NP IN cDCD LT**										
Mohkam et al., 2022 [63]	34 NP cDCD LT	0%	20.0	2.3	/	/	0	*	*	*
	68 NRP cDCD LT	100% 184 min	21.0	5.8	/	/	1.5	*	*	*
Gaurav et al., 2022 [59]	69 NRP cDCD LT	100% 133 min	19.0	6.7	/	/	0	*	*	*
	67 NP cDCD LT	0%	15.0	6.6	/	/	1.5	*	*	*
	97 SCS cDCD LT	0%	15.0	7.2	/	/	5.2	*	*	*

SLKT Simultaneous Liver-Kidney Transplantations; LT: Liver Transplantation; KT: Kidney Transplantation; DBD: Donation after Brain Death; cDCD: Controlled Donation after Circulatory Death; NP: Ex-Situ Normothermic Perfusion; NRP: Normothermic Regional Perfusion; SCS: Static Cold Storage; SRR: Super-Rapid Recovery; HOPE: Ex-Situ Hypothermic Oxygenated Perfusion; ECD: Extended Criteria Donors

**Table 3:** Studies evaluating cDCD SLKT and LT outcomes: patient and graft survival

Author, date	Arms (Number of recipients and donor type)	1-year patient survival (%)	1-year liver allograft survival (%)	1-year kidney allograft survival (%)	3-years patient survival (%)	3-years liver allograft survival (%)	3-years kidney allograft survival (%)
**STUDIES EVALUATING cDCD VERSUS DBD IN SLKT**							
Nunez-Nateras et al., 2020 [45]	30 cDCD SLKT	96.7	93.3	93.3	93.3	90.0	90.0
	131 DBD SLKT	95.4	93.1	93.1	93.3	90.1	90.1
Croome et al., 2020 [46]	94 cDCD SLKT (Era 1)	72.9	69.2	67.4	64.8	59.5	58.4
	208 cDCD SLKT (Era 2)	87.6	83.1	86.5	82.4	76.6	79.4
	624 DBD SLKT (propensity matched) (Era 2)	91.3	90.4	88.6	83.4	82.3	81.5
LaMattina et al., 2011 [47]	5 cDCD SLKT	100	100	100	*	*	*
	32 DBD SLKT	97	94	94	*	*	*
**STUDIES EVALUATING NRP VERSUS SRR IN cDCD LT**							
Hessheimer et al., 2019 [60]	95 NRP cDCD LT	93	88	/	93	88	/
	117 SRR cDCD LT	88	83	/	84	76	/
Watson et al., 2019 [51]	43 NRP cDCD LT	97	98	/	94	93	/
	187 SRR cDCD LT	95	87	/	93	85	/
Hessheimer et al., 2022 [50]	545 NRP cDCD LT	92	90	/	89	87	/
	258 SRR cDCD LT	86	79	/	76	68	/
**STUDIES EVALUATING NRP cDCD VERSUS DBD IN LT**							
Savier et al., 2020 [52]	50 NRP cDCD LT	97	95	/	83	80	/
	100 DBD LT	90	88	/	84	82	/
**STUDIES EVALUATING NP VERSUS SCS IN LT**							
Nasralla et al., 2018 [56]	170 NP LT (107 DBD and 63 cDCD)	96	95	/	*	*	/
	164 SCS LT (104 DBD and 60 cDCD)	97	96	/	*	*	/
**STUDIES EVALUATING HOPE VERSUS SCS IN LT**							
Schlegel et al., 2019 [61]	50 HOPE cDCD LT	98	92	/	94	92	/
	50 SCS cDCD LT	90	88	/	88	80	/
	50 SCS DBD LT	92	88	/	92	88	/
Czigany et al., 2021 [62]	23 HOPE ECD DBD LT	91	91	/	*	*	/
	23 SCS ECD DBD LT	83	78	/	*	*	/
Schlegel et al., 2023 [57]	85 HOPE DBD LT	95.3	95.3	/	*	*	/
	85 SCS DBD LT	95.3	91.8	/	*	*	/
**STUDIES EVALUATING NRP VERSUS HOPE IN cDCD LT**							
Muller et al., 2020 [58]	132 NRP cDCD LT	95	93	/	92	88	/
	93 HOPE cDCD LT	93	86	/	90	83	/
**STUDIES COMPARING NRP VERSUS NP IN cDCD LT**							
Mohkam et al., 2022 [63]	34 NP cDCD LT	94.1	88.2	/	*	*	/
	68 NRP cDCD LT	98.5	93.9	/	*	*	/
Gaurav et al., 2022 [59]	69 NRP cDCD LT	94.0	93.0	/	94.0	90.0	/
	67 NP cDCD LT	94.0	88.0	/	90.0	76.0	/
	97 SCS cDCD LT	94.0	84.0	/	88.0	76.0	/

SLKT Simultaneous Liver-Kidney Transplantations; LT: Liver Transplantation; KT: Kidney Transplantation; DBD: Donation after Brain Death; cDCD: Controlled Donation after Circulatory Death; NP: Ex-Situ Normothermic Perfusion; NRP: Normothermic Regional Perfusion; SCS: Static Cold Storage; SRR: Super-Rapid Recovery; HOPE: Ex-Situ Hypothermic Oxygenated Perfusion; ECD: Extended Criteria Donors

Nunez-Nateras et al. [45] compared outcomes of DBD and cDCD SLKT. From January 2010 to December 2018, they included 30 cDCD SLKT and 131 DBD SLKT, from 2 US centers. Median warm ischemia time (WIT) was 24 min in the cDCD SLKT cohorts. They reported similar kidney DGF rates, similar 1-year patient survival (96.7% vs. 95.4% in cDCD and DBD), similar 1-year liver allograft survival (93.3% vs. 93.1%) and similar 1-year kidney allograft survival (93.3% vs. 93.1%). In their recent study, from UNOS database, Croome et al. [46] compared outcomes of cDCD SLKT performed between 2000 and 2010 and 2011–2018 and confirmed outcomes reported by Nunez-Nateras et al. [45]. Indeed, they reported better outcomes in 2011–2018 period: higher 3-years patient survival, higher 3-years liver allograft survival and higher 3-years kidney allograft survival. LaMattina et al. [47] reported the University of Wisconsin experience on cDCD SLKT and compared outcomes with DBD SLKT. From January 1998 to December 2008, they included 5 cDCD SLKT and 32 DBD SLKT. Median WIT was 19.0 min in cDCD SLKT group. They reported a non-significant higher kidney DGF rate in cDCD SLKT group and similar 1-year kidney and liver allograft survival (Kidney and Liver: 100% versus 94% in cDCD and DBD).

### In-situ and *ex-situ* perfusion strategies to improve organ preservation in LT. Application for SLKT?

Main studies comparing in-situ and *ex-situ* perfusion strategies in LT are summarized in Tables 1, 2 and 3.

Normothermic regional perfusion (NRP), developed from extracorporeal membranous oxygenation (ECMO), was described by Johnson LB et al. in 1997 [48]. This in-situ normothermic perfusion allows to restore metabolic function by restoration of cellular energy substrates and allow to assess the suitability of the organs [49].

Recently, Hessheimer et al. [50] compared outcomes of Maastricht III cDCD liver transplantations performed with postmortem in-situ NRP versus super rapid recovery (SRR). They included 545 cDCD LT with in-situ NRP and 258 with SRR, from June 2012 and December 2019. They reported that NRP was a protective factor for ischaemic biliary complications and graft loss. Watson et al. [51] reported similar outcomes. They compared outcomes of Maastricht III cDCD liver transplantations performed with postmortem in-situ NRP versus SRR. From January 2011 to June 2017, they included 43 cDCD LT with in-situ NRP and 187 with SRR, in all UK centers. Median NRP duration was 123 min. They reported that NRP was associated with a reduction in 30-day graft loss, ischaemic biliary complications and anastomotic strictures.

In France, Savier et al. [52] compared outcomes of cDCD LT with in-situ NRP and DBD LT. They included 50 cDCD with in-situ NRP and 100 DBD. They reported similar arterial complications, biliary complications, 2-years graft survival and 2-years patient survival.

Moreover, *ex-situ* preservation is of paramount importance in the context of cDCD SLKT. Since the development of hypothermic machine perfusion by Alexis Carrel over a century ago [53], this preservation method has grown in its utility, initially in kidney transplantation. Indeed, many studies have demonstrated the benefit of HMP in kidney transplantation, particularly for its significant reduction of the incidence of delayed graft function for DCD and ECD [54].

In liver transplantation, in order to limit the negative effects of WIT, 2 main dynamic preservation strategies have been developed in clinical practice: *ex-situ* normothermic perfusion (NP) and *ex-situ* hypothermic oxygenated perfusion (HOPE) [55].

In 2018, Nasralla et al. [56] reported outcomes of the first randomized trial comparing conventional static cold storage (SCS) and *ex-situ* NP for liver transplantation. They included 170 NP liver transplantation and 164 SCS liver transplantation. The proportion of DBD were 62.9% and 63.4% in NP and SCS groups. They reported a significant decrease on peak AST (primary outcomes) of 49.4% and a significant decrease of early allograft dysfunction of 74% in NP group. They reported similar biliary complications, graft and patient survival between NP and SCS groups.

Recently, Schlegel et al. [57] compared outcomes of DBD LT with *ex-situ* HOPE and DBD LT with conventional SCS. From 2015 to 2019, they included 85 DBD LT with *ex-situ* HOPE and 85 DBD LT with conventional SCS. They reported similar major post-operative complications (Clavien ≥ III).

Currently, no study compared *ex-situ* normothermic perfusion (NP) and *ex-situ* hypothermic oxygenated perfusion (HOPE) for LT.

Muller et al. [58] compared outcomes of cDCD LT after in-situ NRP and *ex-situ* HOPE. They included 132 cDCD LT with in-situ NRP and 93 cDCD LT with *ex-situ* HOPE. They reported similar biliary complications rates, arterial thrombosis rates, primary non-function rates, 1-year graft and patient survival.

Recently, Gaurav et al. [59] reported outcomes of cDCD after in-situ NRP, cDCD after *ex-situ* NP and cDCD after conventional SCS. They reported a better early liver function using in-situ NRP and *ex-situ* NP, compared to conventional SCS.

## Discussion

Some limitations of this narrative review should be mentioned. Firstly, the main limitation is the low level of included studies, the heterogeneity of evaluation between studies and the paucity of data. Indeed, several data were poorly reported, in particular mid-term patient survival and graft survival in studies evaluating DBD and DCD and in-situ and *ex-situ* preservation strategies.

## Conclusion

Simultaneous liver-kidney transplantation has made a significant contribution for patients with dual-organ disease. The optimization of indication and selection of SLKT patients will reduce futile transplantation. Moreover, increasing the use of transplants from extended criteria donors, in particular cDCD, should be encouraged, thanks to the development of new modalities of organ preservation.

### Author contributions

Thomas PRUDHOMME: Protocol development, Data collection, Data analysis, Manuscript writing Benoit MESNARD: Protocol development, Data collection, Data analysis, Manuscript writing Julien BRANCHEREAU: Protocol development, Manuscript writing Mathieu ROUMIGUIÉ: Data analysis, Manuscript editing Charlotte MAULAT: Data analysis, Manuscript editing Fabrice MUSCARI: Data analysis, Manuscript editing Nassim KAMAR: Data analysis, Manuscript editing Michel SOULIÉ: Data analysis, Manuscript editing Xavier GAMÉ: Data analysis, Manuscript editing Federico SALLUSTO: Data analysis, Manuscript editing Marc Olivier TIMSIT: Protocol development, Data collection, Data analysis, Manuscript editing Sarah DROUIN: Protocol development, Data collection, Data analysis, Manuscript writing and editing.

## Electronic supplementary material

Below is the link to the electronic supplementary material.

Supplementary Material 1

Supplementary Material 2

## **Abbreviations**

CNI: Calcineurin Inhibitors
DBD: Donation after Brain Death
DCD: Donation after Circulatory Death
DGF: Delayed Graft Function
DSA: Donor-Specific Anti-HLA Antibodies
eGFR: Estimated Glomerular Filtration Rate
ECD: Extended Criteria Donors
ESLD: End-Stage Liver Disease
ESRD: End-Stage Renal Disease
HCV: Hepatitis C Virus
HMP: Hypothermic Machine Perfusion
HOPE: Ex-Situ Hypothermic Oxygenated Perfusion
HRS: Hepatorenal Syndrome
KALT: Kidney After Liver Transplantation
KT: Kidney Transplantation
LT: Liver Transplantation
MELD: Model for End-Stage Liver Disease
NP: Ex-Situ Normothermic Perfusion
NRP: Normothermic Regional Perfusion
OPTN: Organ Procurement and Transplantation Network
PNF: Primary Non-Function
SCS: Static Cold Storage
SLKT: Simultaneous Liver-Kidney Transplantations
SRR: Super-Rapid Recovery
UNOS: United Network for Organ Sharing
WIT: Warm Ischemia Time
