# Supplementary material for: Simultaneous liver-kidney transplantation: future perspective
Source: World J Urol. 2024 Aug 20;42(1):489. doi: 10.1007/s00345-024-05174-z (PMC11335780; doi:10.1007/s00345-024-05174-z)
Supplement: Supplementary file 1 — Supplementary Material 1 [file 345_2024_5174_MOESM1_ESM.docx]

**Research strategy:**

Pubmed search strategy: ((((((kidney [tf] OR renal [tf]) AND (hepatic [tf] OR liver [tf])) OR hepatorenal [tf])) AND ((Simultaneous* [tiab] OR slkt [tiab] OR slk [tiab] OR combined [tiab] OR combination [tiab] OR concurrent* [tiab] OR sequential* [tiab]) AND (Graft* [tiab] OR allograft* [tiab] OR allogeneic [tiab] OR homologous* [tiab] OR homograft* [tiab] OR transplant* [tiab] OR Transplants [mh] OR Transplantation [mh])))) OR (((((Kidney Transplantation [mh] OR Kidney/transplantation [mh]) AND (Liver Transplantation [mh] OR Liver/transplantation [mh])) AND (Simultaneous* [tiab] OR slkt [tiab] OR slk [tiab] OR combined [tiab] OR combination [tiab] OR concurrent* [tiab] OR sequential* [tiab]))) OR ((“liver and kidney” [tiab] OR “kidney and liver” [tiab] OR “liver kidney” [tiab] OR “kidney liver” [tiab]) AND (Simultaneous* [tiab] OR slkt [tiab] OR slk [tiab] OR combined [tiab] OR combination [tiab] OR concurrent* [tiab] OR sequential* [tiab]) AND (Graft* [tf] OR allograft* [tf] OR allogeneic [tf] OR homologous* [tf] OR homograft* [tf] OR transplant* [tf] OR Transplants [mh] OR Transplantation [mh]))).
