# Supplementary material for: Simultaneous liver-kidney transplantation: future perspective
Source: World J Urol. 2024 Aug 20;42(1):489. doi: 10.1007/s00345-024-05174-z (PMC11335780; doi:10.1007/s00345-024-05174-z)
Supplement: Supplementary file 2 — Supplementary Material 2 [file 345_2024_5174_MOESM2_ESM.docx]

**Supplementary Figure 1 -** PRISMA Flow Chart

Records removed *before screening*:

Duplicate records removed (n = 731)

Records identified through database searching

(n = 1,917)

**Identification**

Records excluded

(n = 1,107)

Abstract only (n = 631)

Case reports (n= 122)

Irrelevant studies (n= 354)

**Screening**

Records screened

(n = 1,186)

Full-text articles assessed for eligibility

(n = 79)

**Eligibility**

Records excluded (n = 21)

Invalid population (n = 10)

Publication before 1995 (n = 11)

Studies included

(n = 58)

**Included**
